# Polymer Chain Mobility under Shear—A Rheo-NMR Investigation

**DOI:** 10.3390/polym10111231

**Published:** 2018-11-07

**Authors:** Brigitte Wiesner, Benjamin Kohn, Mandy Mende, Ulrich Scheler

**Affiliations:** Leibniz-Institut für Polymerforschung Dresden e.V., Hohe Str. 6, 01069 Dresden, Germany; Brigitte.Wiesner@mail.com (B.W.); kohn@ipfdd.de (B.K.); mandy.mende66@gmail.com (M.M.)

**Keywords:** polymer mobility, rheo NMR, entanglements

## Abstract

The local dynamics in polymer melts and the impact of external shear in a Couette geometry have been investigated using rheological nuclear magnetic resonance (NMR). The spin-spin relaxation time, T_2_, which is sensitive to chain-segment motion, has been measured as a function of shear rate for two samples of poly(dimethylsiloxane). For the low-molecular-weight sample, a mono-exponential decay is observed, which becomes slightly faster with shear, indicating restrictions of the polymer chain motion. For the high-weight sample, a much faster bi-exponential decay is observed, indicative of entanglements. Both components in this decay become longer with shear. This implies that the free polymer segments between entanglements become effectively longer as a result of shear.

## 1. Introduction

The dynamics and ordering of macromolecules under shear is of general interest for the understanding of the dynamic behavior as well as in the processing of polymer materials; a combination of both orientation and deformation of the polymer is expected [1]. Common rheological experiments measure the global effects [2,3]. Recently, combinations with other methods, like scattering, have successfully been introduced [4,5], providing some insight into the molecular behavior under shear which may be applied in continuous or oscillatory fashion.

The combination of various nuclear magnetic resonance (NMR) experiments with flow or shear, termed rheo-NMR, is one intriguing way to obtain non-destructive insight into flow pattern or into molecular information of materials under shear and flow conditions [6]. It has been used to measure flow pattern [7] or to derive molecular parameters which reflect molecular structure or dynamics [8]. A variety of parameters is available to generate contrast in images [9] or for quantitative analysis in materials science. Besides materials studies and polymer physics, there have also been recent applications to proteins [10], including an improved understanding of the formation of silk fibers [11].

A typical property of polymers is the presence of entanglements, which manifest themselves in heterogeneity of the polymer mobility which can be observed in relaxation NMR and other methods. Transverse or spin-spin relaxation (T_2_) has been widely used to characterize polymer chain mobility, because it is sensitive to the slow motions of polymer chain segments [12,13]. In systems containing a high concentration of protons, the transverse relaxation is strongly affected by the residual dipolar coupling between the protons, which is partially averaged by molecular motion. In general, more free motion or longer chain segments between crosslinks and entanglements (physical crosslinks) leads to a longer T_2_ because of the lowered residual dipolar couplings. The basis of the present investigation is the shear-induced changes of T_2_.

To investigate the effects discussed above, two samples of poly(dimethylsiloxane) of relatively narrow molecular weight distribution has been investigated under shear in situ. 

## 2. Materials and Methods 

The experiments have been performed on a Bruker Avance I NMR spectrometer (Bruker BioSpin, Karlsruhe, Germany) using a 7 T widebore magnet operating at a Larmor frequency of 300 MHz for protons. The spectrometer is equipped with a Bruker Micro 2.5 microimaging system with three orthogonal gradients of 1 T/m maximum gradient strength for each channel. This is applied to control the adjustment of the Couette cell and to check the flow profile using flow NMR using the same cell in an imaging probe head with a birdcage resonator with 10 mm inner diameter. For better sensitivity, linewidth and radio-frequency performance, a 10 mm Bruker high-resolution NMR probe head has been used in conjunction with an in-house-built rheo-NMR accessory. In the 10-mm NMR tube, a 7.7 mm rotor made from Vespel has been inserted. This rotor is connected to a drive shaft inserted from the top of the magnet containing ceramic ball bearings for smooth and centric rotation. From the top the shaft is driven by a servomotor, at a rotation rate which is controlled from an in-house-built rheo-NMR controller, which had been used previously for high-temperature investigations [14].

To measure the transverse relaxation time, T_2_, the Carr-Purcell-Meibom-Gill (CPMG) sequence has been applied [15,16]. This multi-echo sequence permits measuring the relaxation decay without any modulation from diffusion and J coupling. The CPMG pulse sequence is, to a large extent, compensated for radio-frequency pulse imperfections, which is important considering the large number of echo pulses applied here. Between 2 and 1500 echoes have been recorded with an echo delay of 1 ms and a 50 μs π pulse duration. To ensure a constant temperature, the probe head and the sample have been in a constant air flow at room temperature.

Poly(dimethylsiloxane) (PDMS) samples of 410 g/mol and 63,000 g/mol have been purchased from Alfa Aesar (Karlsruhe, Germany) and used as received without any further purification or treatment. According to the supplier, the materials exhibit a polydispersity below 1.15. After inserting the samples in the Couette cell, they were weakly stirred to remove any possible air bubbles and then rested for at least 30 min before the start of the experiments. 

## 3. Results

**Figure 1:**
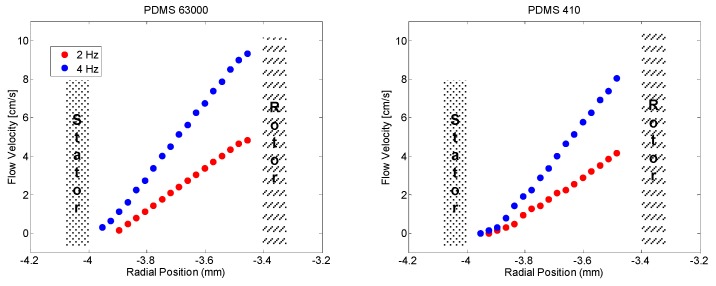
Velocity profiles across the gap in the Couette cell for two velocities for the two samples extracted from three-dimensional flow NMR data.

Flow profiles for the two samples that had been investigated are depicted in Figure 1. The profiles are extracted as a radial slice out of a three-dimensional flow-NMR experiment with one flow-encoding (64 slices) dimension and two space-encoding dimensions, one phase encoded (32 slices) and one frequency encoded (256 complex points), with an echo time of 7.6 ms, read gradient strength of 0.78 T/m, maximal flow-encoding gradient strength of 0.57 T/m resulting in a field of view of 15 mm, and a field of flow of 80 cm/s. In all cases no deviation from a linear flow profile across the radius in the Couette cell has been observed. 

**Figure 2:**
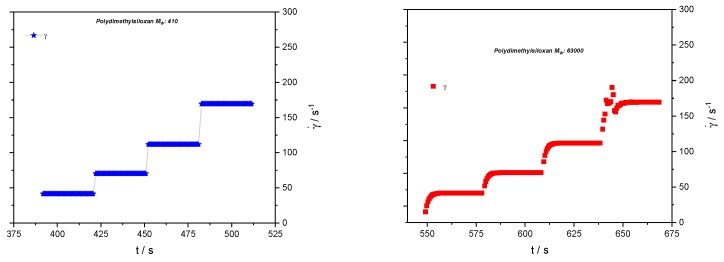
Shear-rate step experiments for the two samples with shear rates subsequently applied in the rheo-NMR experiments.

Rheological tests have been performed on a Haake MARS II rheometer (Thermo Fisher Scientific, Karlsruhe, Germany) applying a cone-and-plate geometry with a gap of 0.034 mm. No shear-rate dependence of the viscosity has been observed for either sample; the viscosities are 1.8 mPa·s and 9470 mPa·s for the low-molecular weight and high-molecular weight samples, respectively. In shear-step experiments, relaxation effects have been observed for the sample of 63,000 g/mol only, as can be seen in Figure 2. 

**Figure 3:**
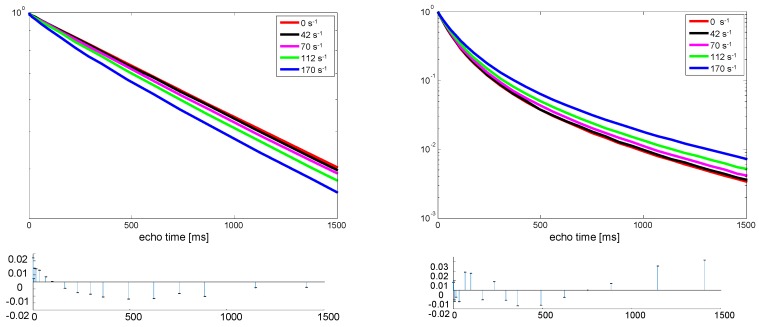
Results from Carr-Purcell-Meibom-Gill (CPMG) measurements of PDMS melts under shear. The insets indicate the shear rates applied in the Couette cell. On the left, the result for the sample of a molecular weight of 410 g/mole, and, on the right, the result for the sample of a molecular weight of 63,000 g/mol, are depicted. Below each figure the residuals from a mono exponential (**left**) or bi-exponential (**right**) fit to the experimental data acquired under the highest shear rate are depicted as a measure of the quality of the fit.

In Figure 3 the signal intensity as a function of the effective echo time (echo time multiplied by the number of echoes) is depicted for two samples of PDMS with different molecular weight. The first significant difference between the two samples is that the spin-spin relaxation time, T_2_, is more than one order of magnitude longer for the sample of low molecular weight, indicating much more molecular motion. This sample is more of an oligomer. The other significant difference is that the low-molecular-weight sample exhibits a mono-exponential decay indicative of homogeneous molecular mobility. In contrast, the high-molecular-weight sample exhibits a bi-exponential decay, clearly showing heterogeneity of the molecular motion, which is explained by the presence of entanglements. The molecular weight is well above the reported entanglement molecular weight of PDMS of about 35,000 g/mol [17]. Entanglements of the polymers leads to restricted motion and thus less averaging of the dipolar coupling and, therefore, shorter T_2_.

Besides the fact that the low-molecular-weight sample exhibits a mono-exponential decay and the high-molecular-weight sample exhibits a bi-exponential decay, there is an opposite trend in the effect of shear. For the low-molecular-weight sample, a slight shortening of T_2_ is observed, while for the sample of higher molecular weight, a significant extension of the decay is observed. In Table 1 the results of a mono-exponential fit for the low-molecular-weight sample and a bi-exponential fit for the sample of higher molecular weight are summarized. These exponential fits describe the experimental results very well; no extra components are required, as can be seen from the residues plotted in Figure 3. The uncertainty in the fit is lower than the last digit given in Table 1. While the relaxation times change, the respective fractions of fast and slow relaxation remained unchanged. Directly after the shear, the samples showed almost the same relaxation times as before shearing, which indicates that the sample temperature did not change significantly.

## 4. Discussion

The slight reduction of T_2_ for the low-molecular-weight sample, which does apparently not exhibit any entanglements, indicates that the polymer chains align under the shear present, which leads to a closer packing of the polymer chains and thus restricted mobility of chain segments. On the other hand, for the sample in which signatures of entanglements are observed, T_2_ increases, showing that there is more polymer chain mobility; the chain segments, which can freely move, become longer. Two scenarios are feasible: either polymer chains are pulled out of entanglements or entanglements are squeezed together and become more localized. Both scenarios lead to effectively longer chain segments between the entanglements and thus an overall larger mobility. However, as there is no change in the intensity fractions for the two components observed, the fraction of entangled polymer remains constant and thus the second scenario describes the finding better. This will be the subject of further investigations.

## 5. Conclusions

Rheo-NMR has been applied as a measure of polymer chain mobility to investigate the effect of continuous external shear on the polymer chain dynamics. The spin-spin relaxation time, T_2_, has been observed for two samples of different molecular weight as a function of the shear rate applied in a Couette cell. A low-molecular-weight sample shows a shortening of T_2_ that can be interpreted as chain ordering. This effect is not observed for a polymer of high molecular weight exhibiting signatures of entanglements. In that case, with shear an increase of the chain mobility is concluded from the NMR relaxation. The fact that the relative amplitudes of the two relaxation components remain unchanged implies that the entanglements become more localized and the effective chain segments, which can move freely, become longer. The chain ordering as observed for the low-molecular-weight sample is not excluded, however the dominating effect for the chain dynamics is the larger chain segments moving freely.

## Figures and Tables

**Table 1:** Summary of the multi-exponential fits to the decay curves. For the bi-exponential decay the respective fractions are quoted in brackets.

Molecular Weight Number of Repeat Units		*M*_W_ 410 5.5	*M*_W_ 63,000 850
Frequency [Hz]	Shear Rate [1/s]	T2 [s]	T2 [s]
0	0	1689	253 (0.3) + 57 (0.7)
2.6	42	1661	263 (0.3) + 61 (0.7)
4.4	70	1635	293 (0.3) + 65 (0.7)
7.0	112	1572	288 (0.3) + 66 (0.7)
10.6	170	1470	319 (0.3) + 73 (0.7)
After Shear	0	1657	279 (0.,3) + 65 (0.7)
